# Supplementary figures and images for: Drug resistance mechanisms create targetable proteostatic vulnerabilities in Her2+ breast cancers
Source: PLoS One. 2022 Dec 8;17(12):e0256788. doi: 10.1371/journal.pone.0256788 (PMC9731458; doi:10.1371/journal.pone.0256788)

Supplementary Figure 1

A

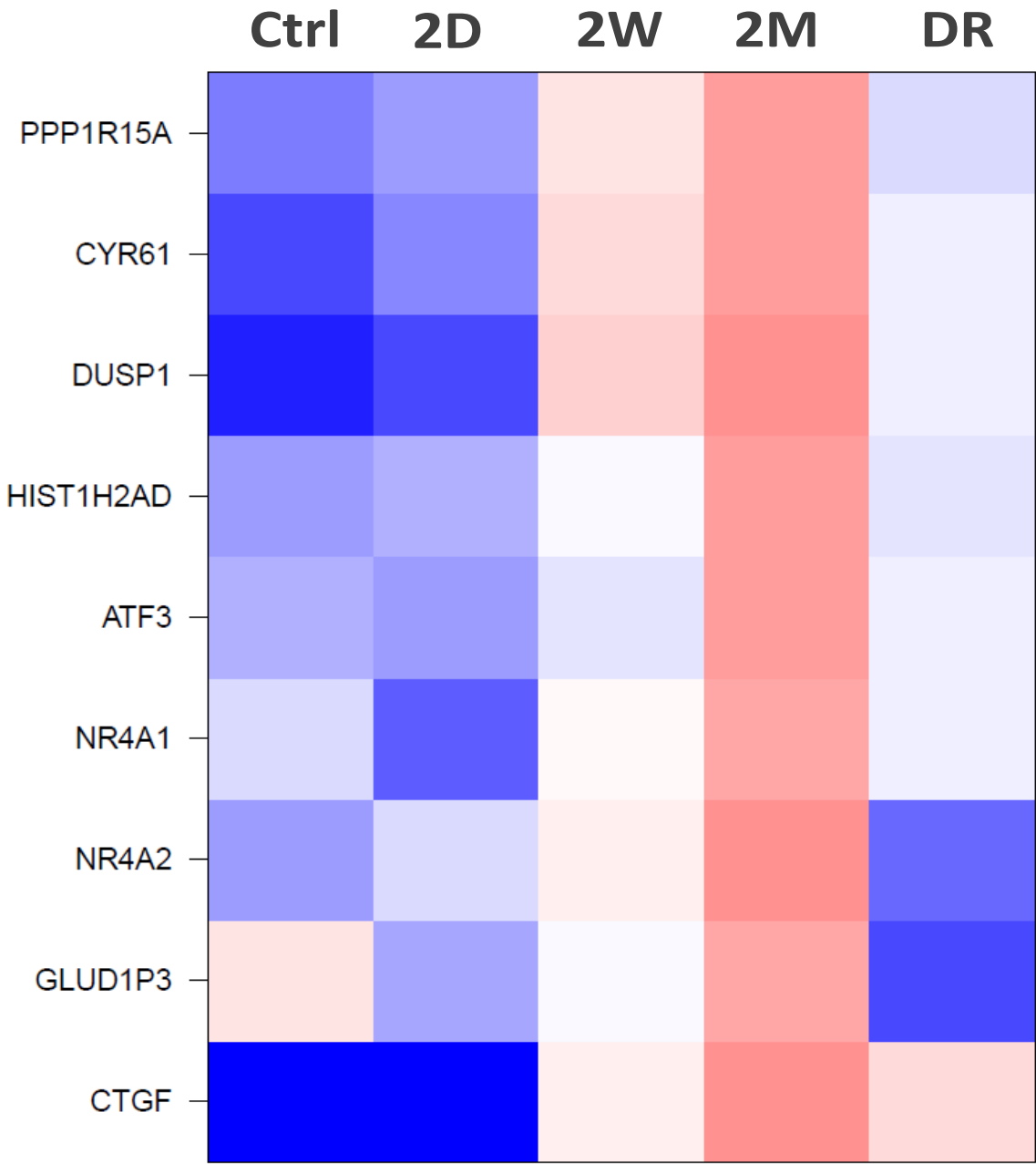

B

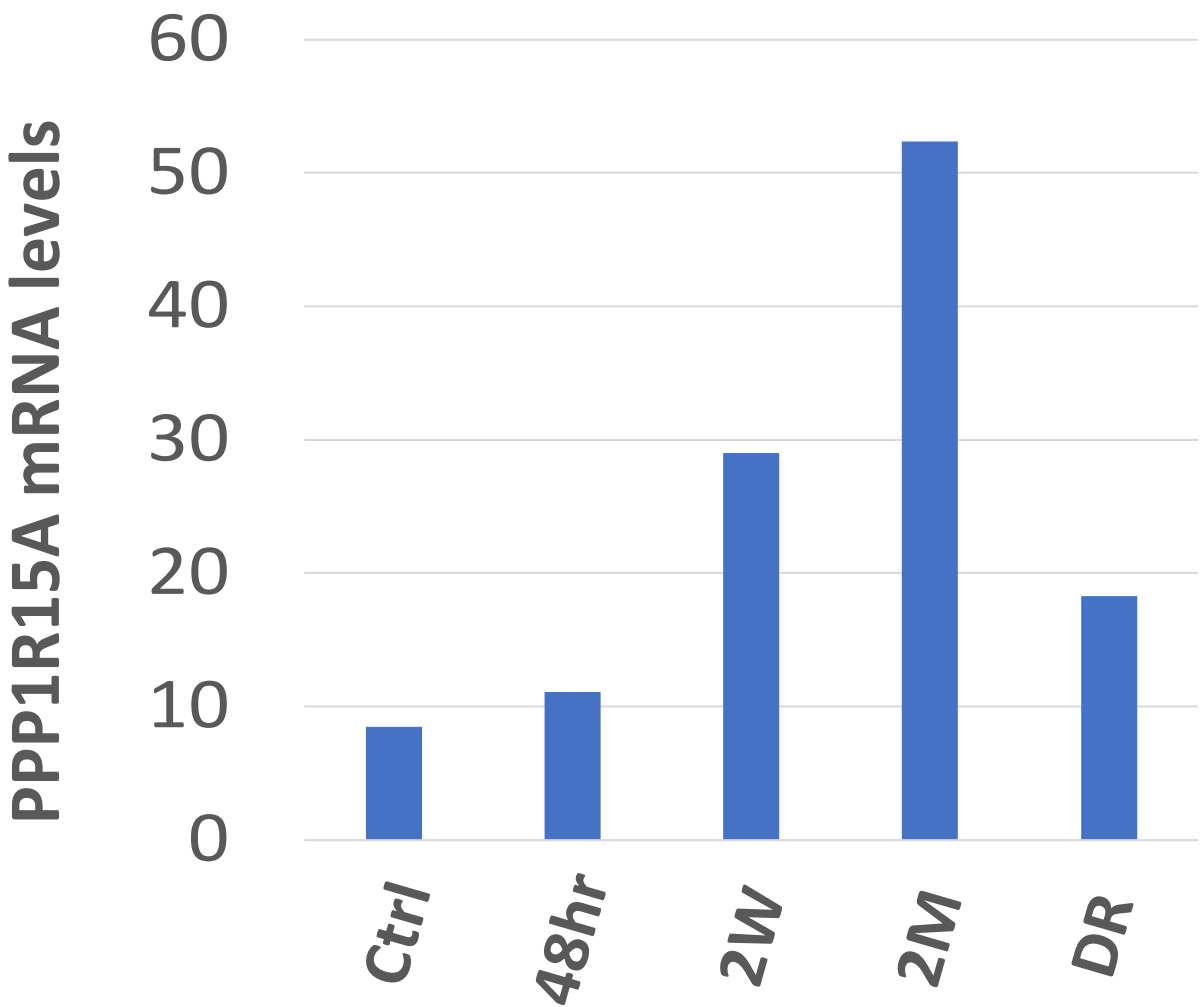

Supplement: S1 Fig — A) heatmap of top genes whose expression was increased in the relapse (2M) samples (from RNAseq). B) Bar plot of expression values for PPP1R15A (from RNAseq) in the indicated samples. (PDF) [file pone.0256788.s003.pdf]

A

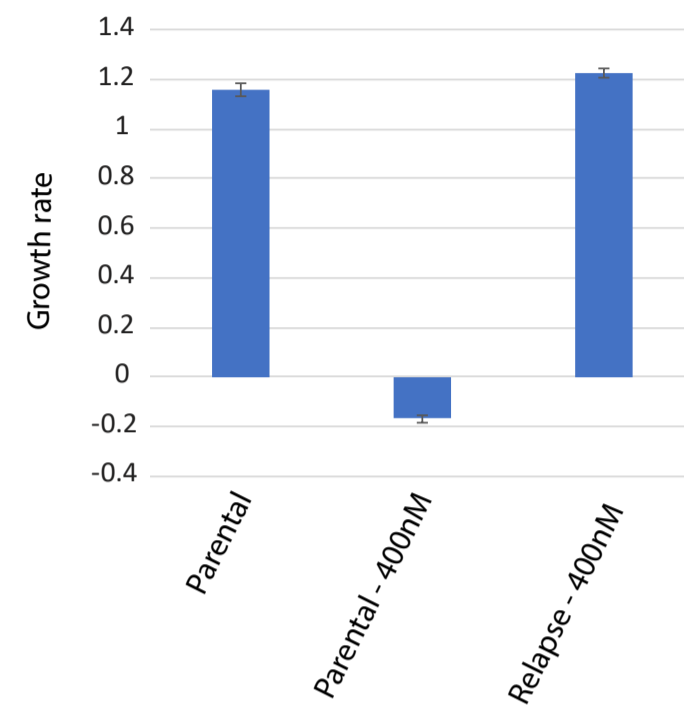

B

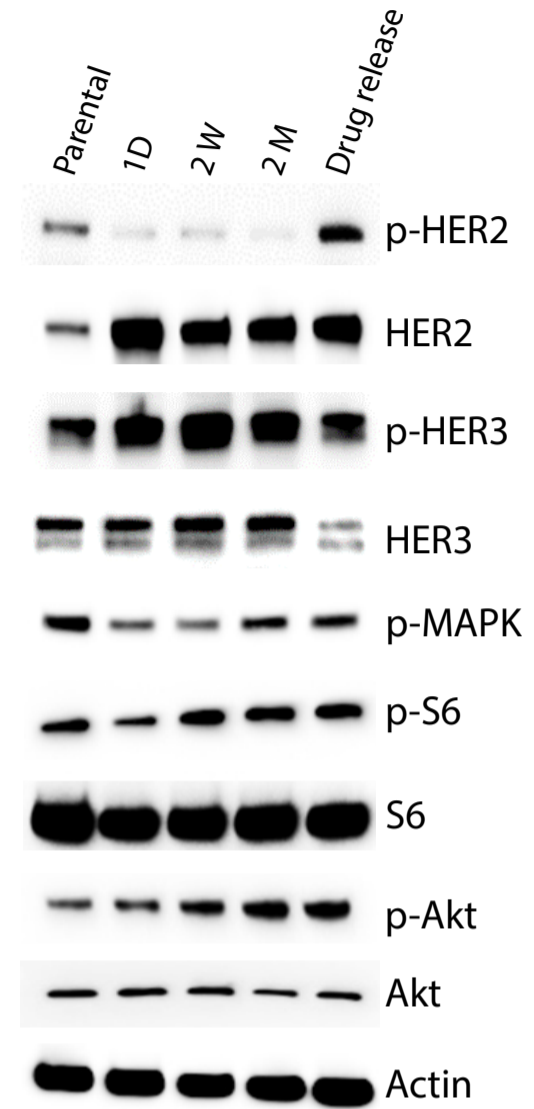

C

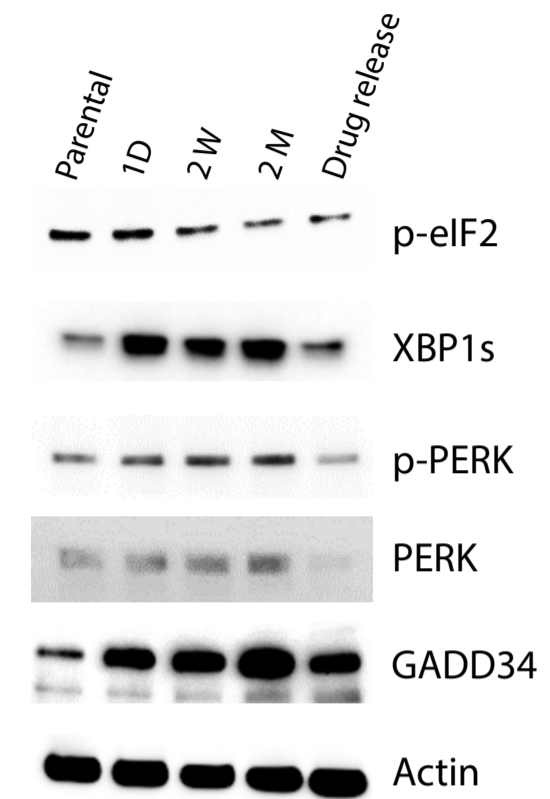

D

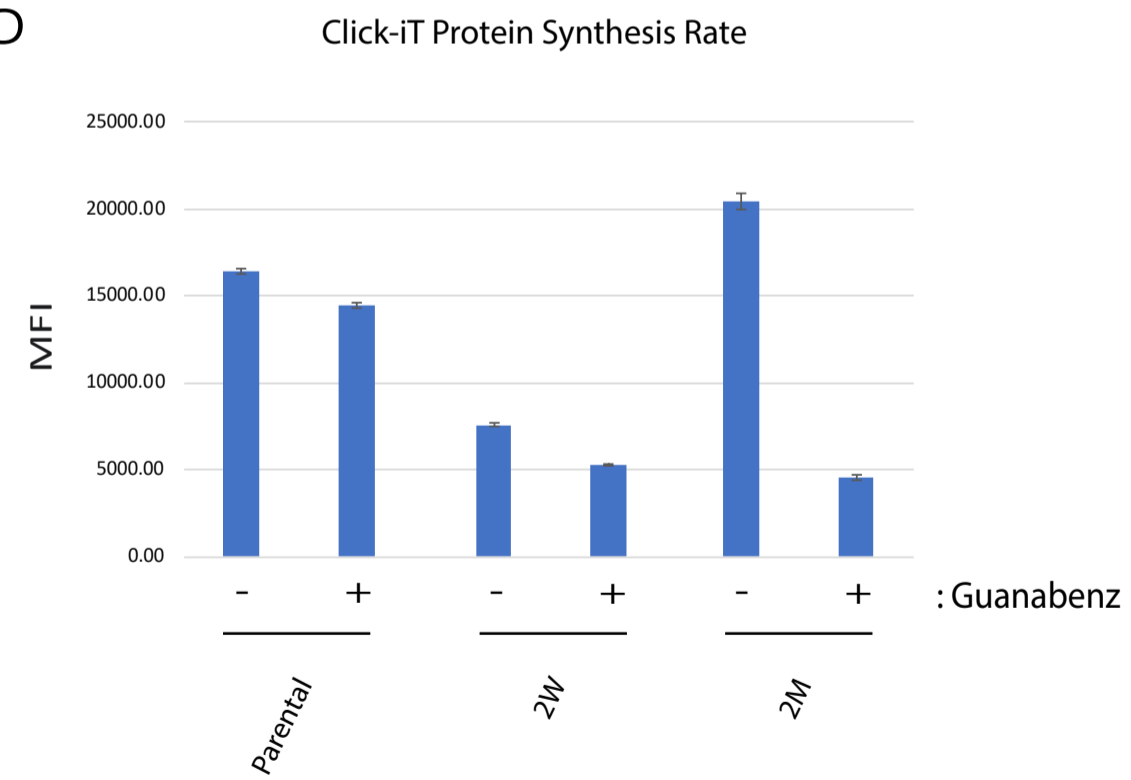

E

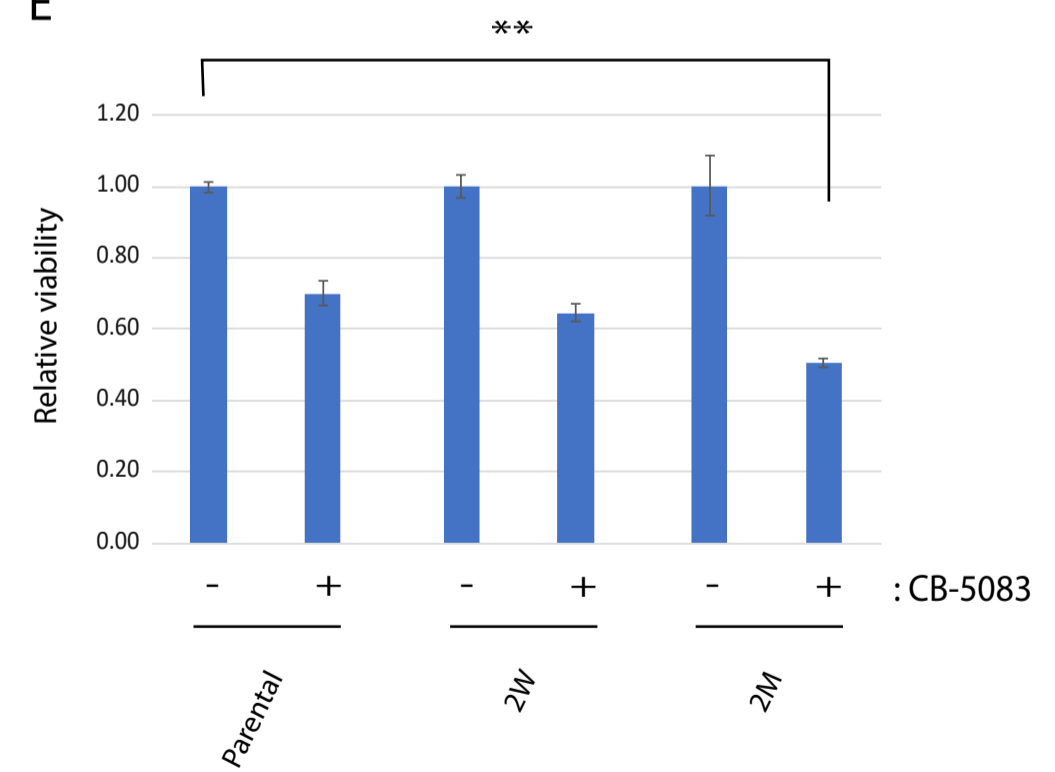

Supplement: S2 Fig — (PDF) [file pone.0256788.s004.pdf]

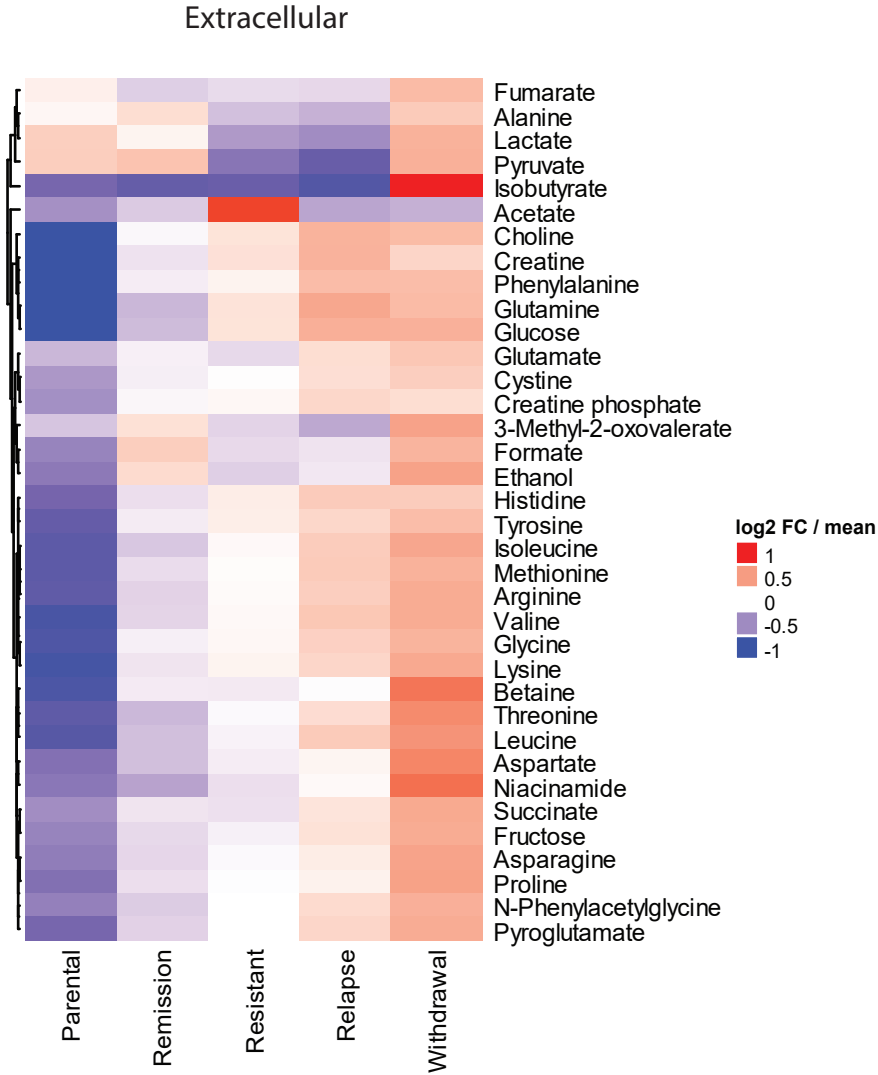

Supplement: S3 Fig — (PDF) [file pone.0256788.s005.pdf]
