# Supplementary material for: Drug resistance mechanisms create targetable proteostatic vulnerabilities in Her2+ breast cancers
Source: PLoS One. 2022 Dec 8;17(12):e0256788. doi: 10.1371/journal.pone.0256788 (PMC9731458; doi:10.1371/journal.pone.0256788)

Supplementary Figure 4

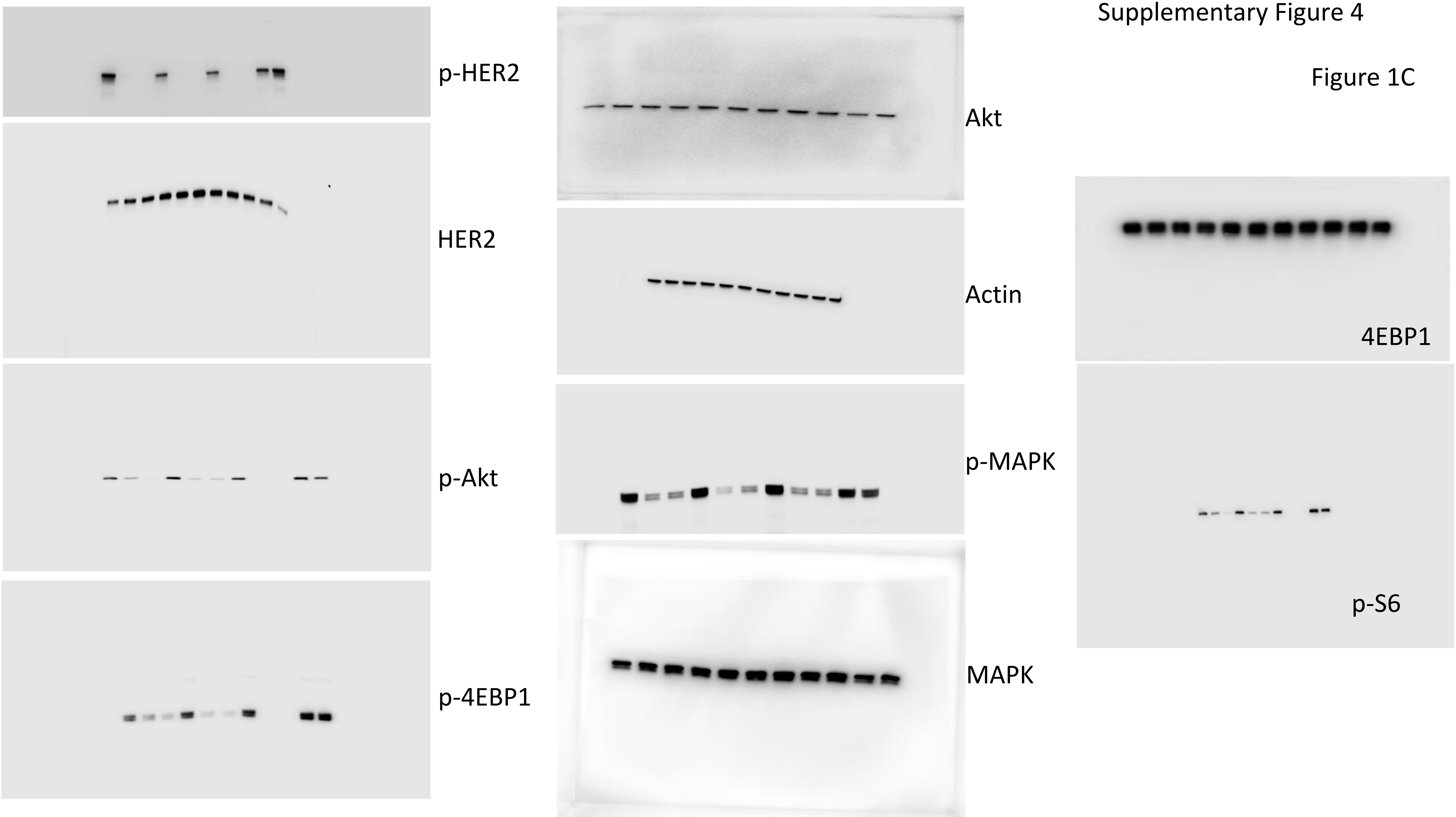

Figure 2

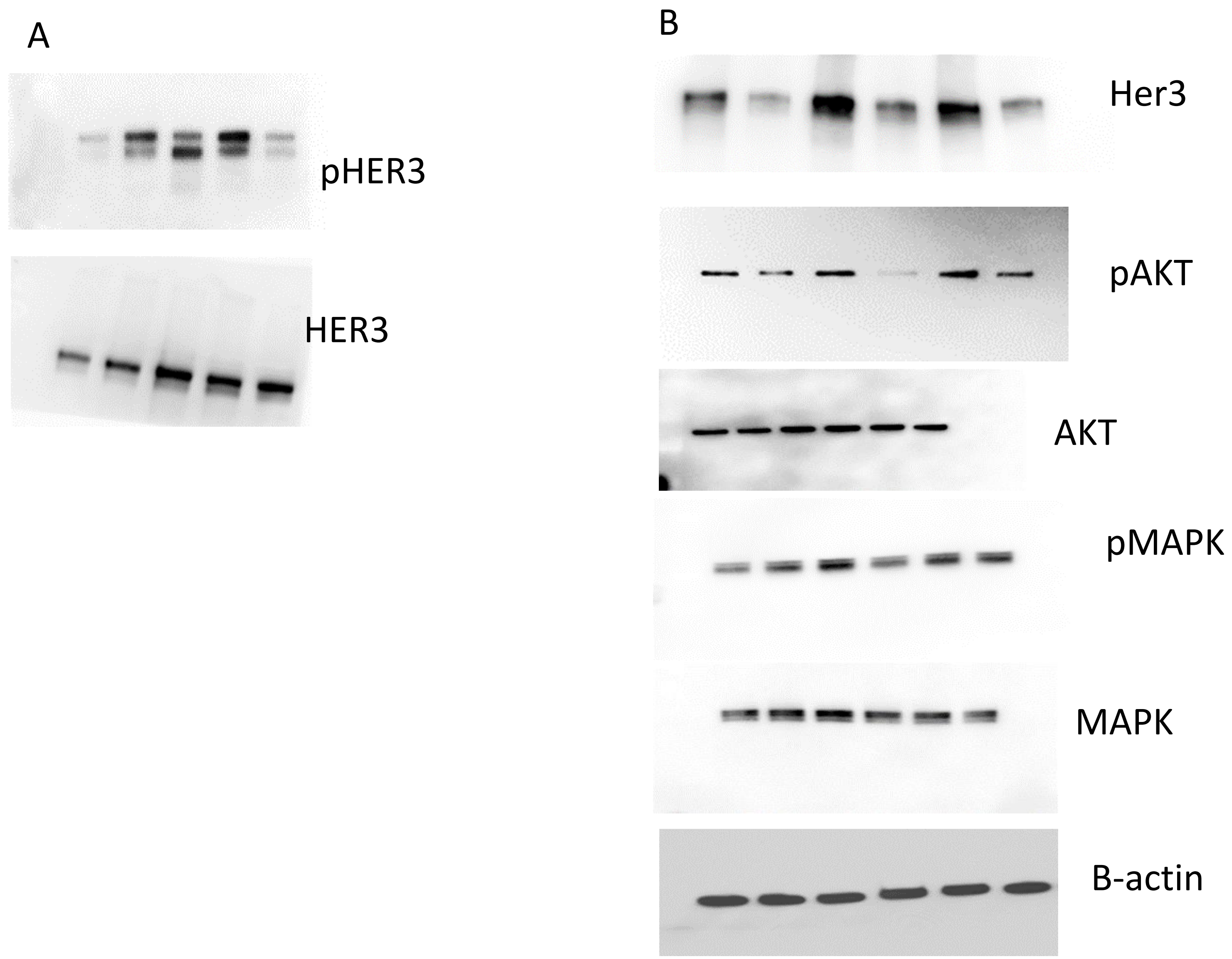

Figure 3D

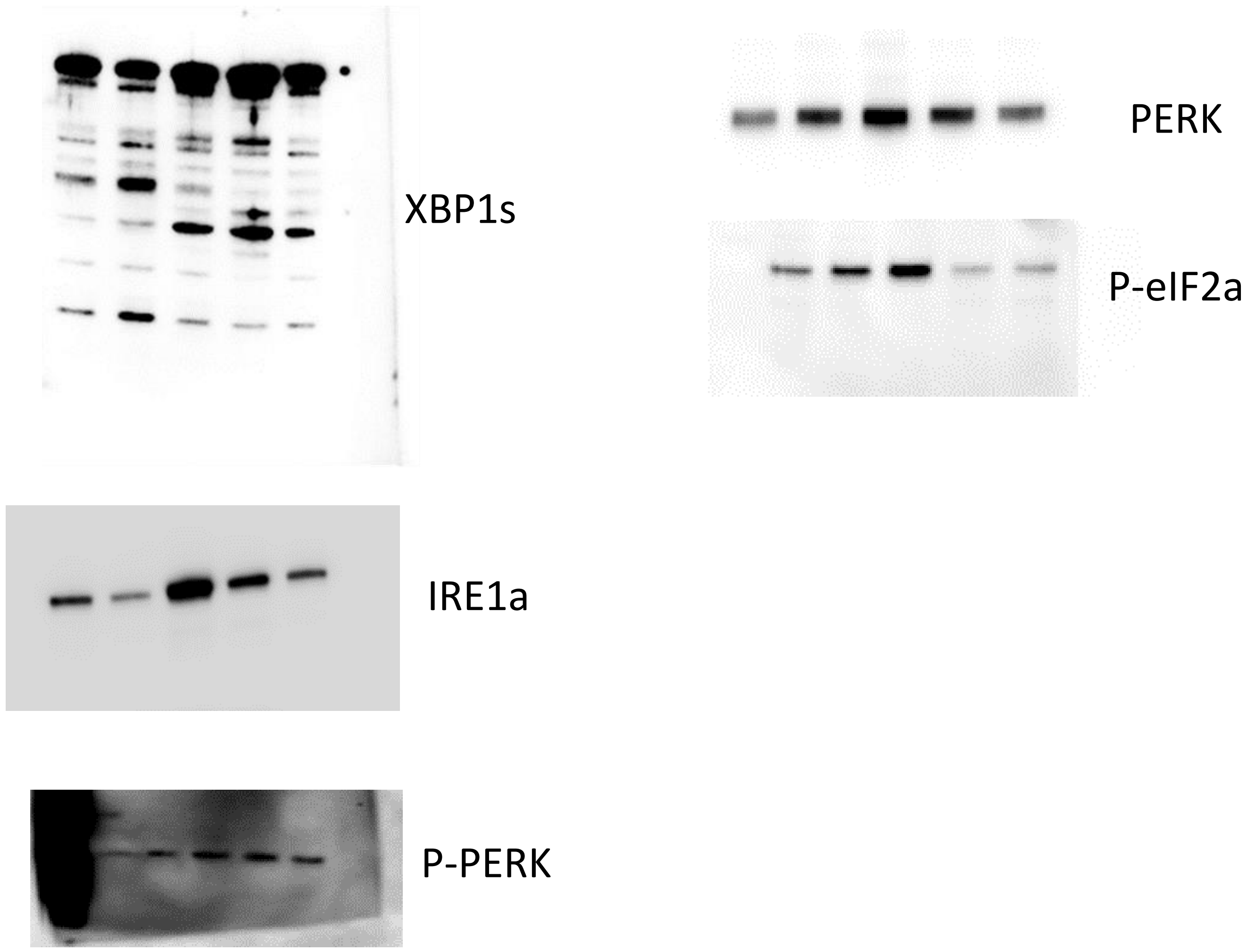

Figure 4C

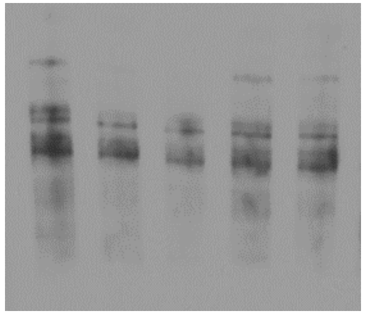

O-GlnNAc

Figure 5

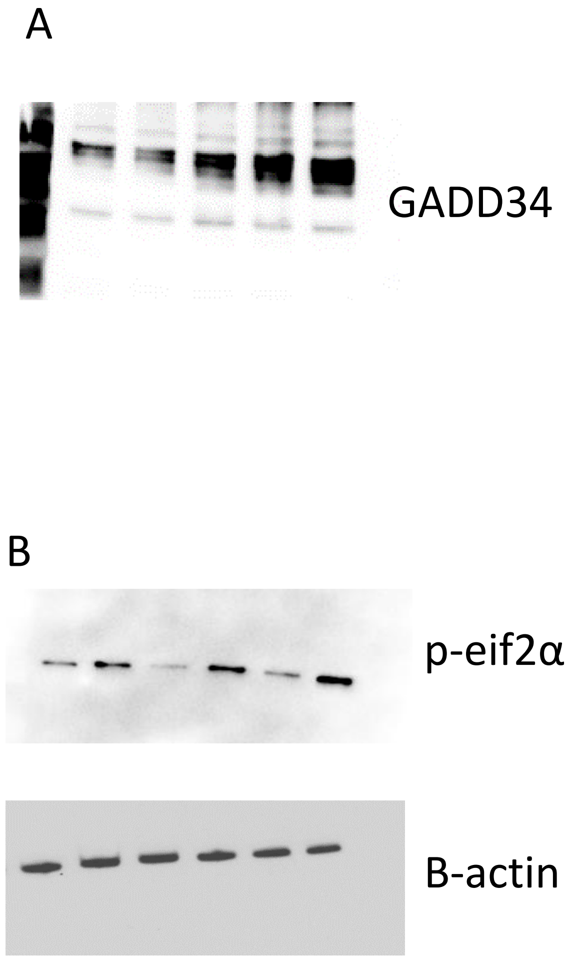

Figure 6

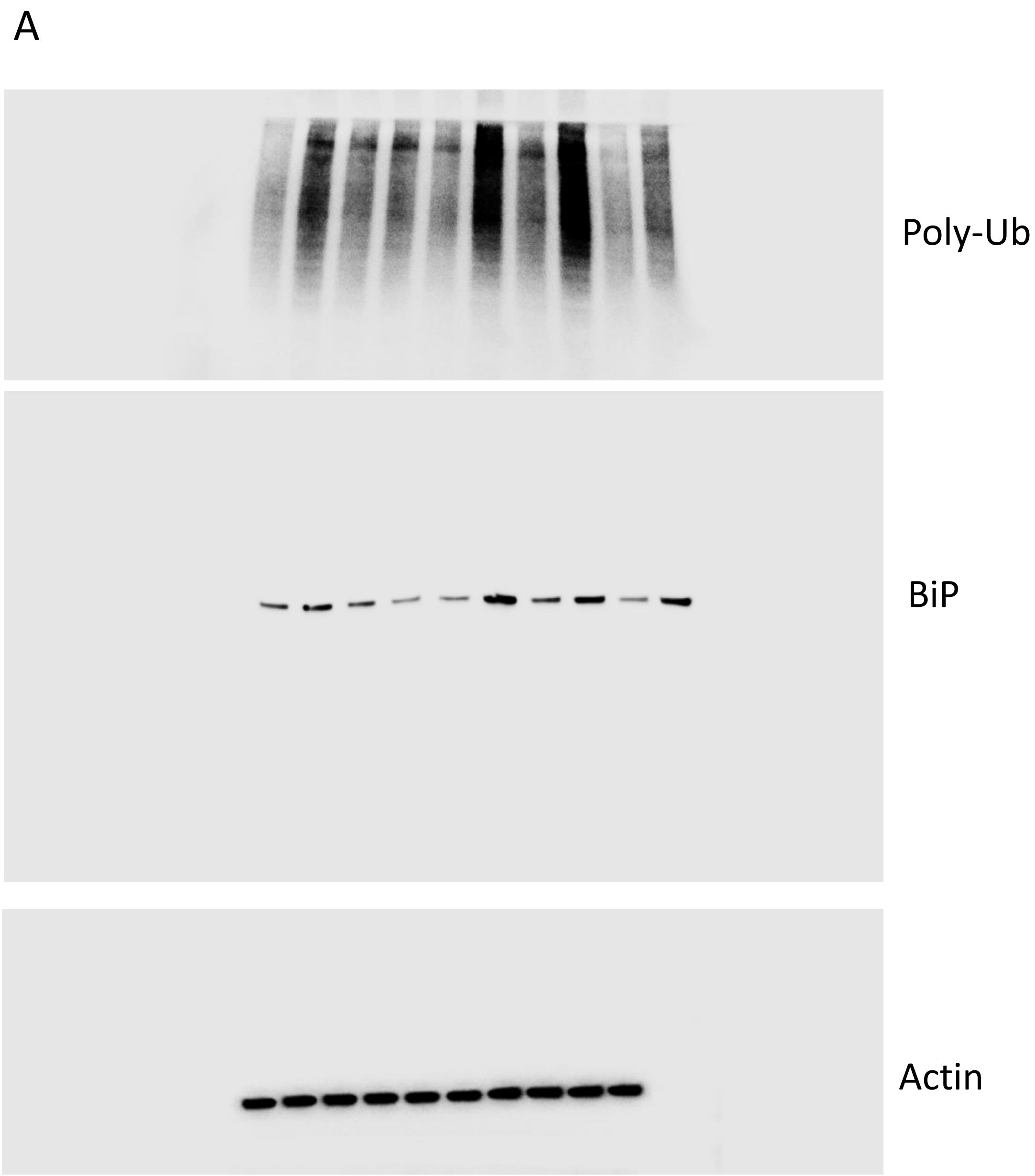

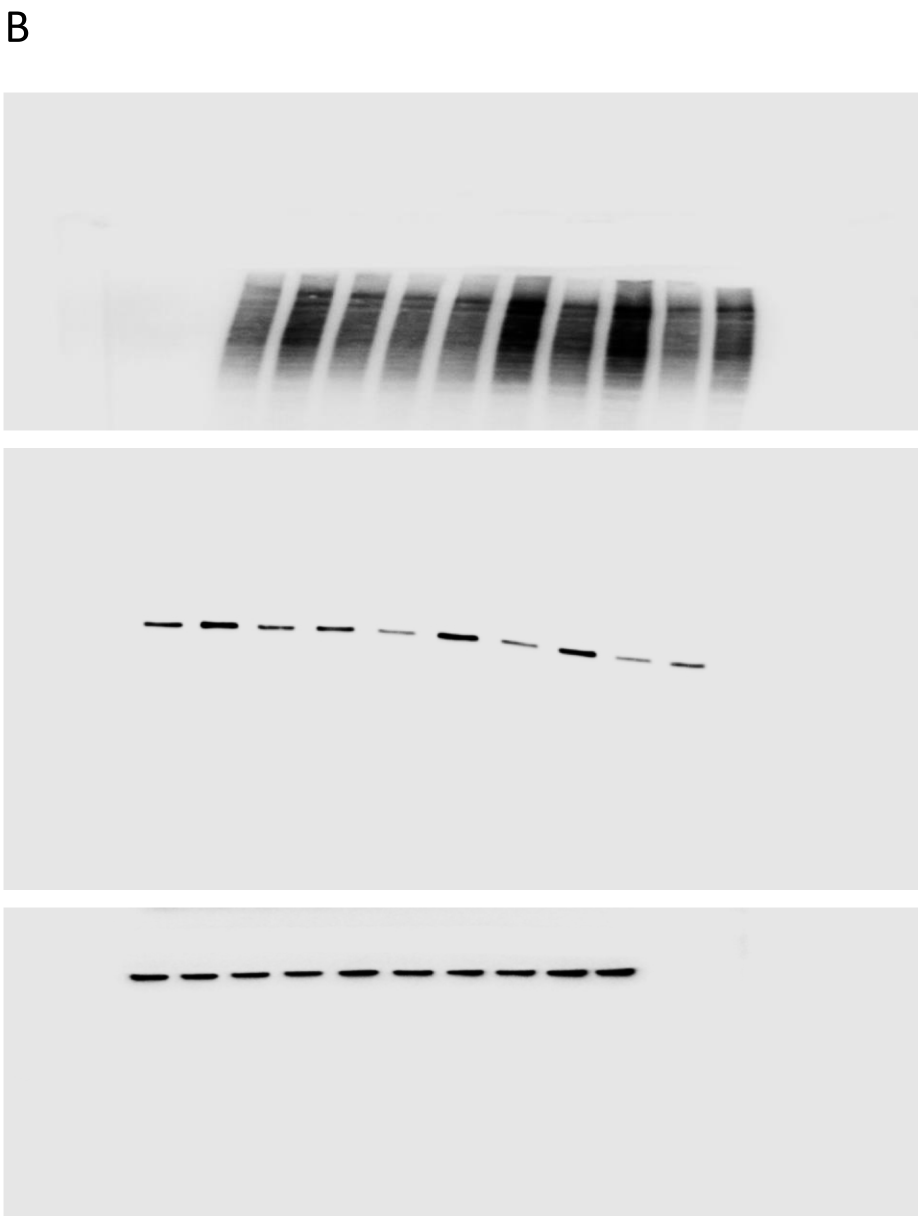

Supplement: S1 Raw images — (PDF) [file pone.0256788.s006.pdf]
