# Supplementary material for: Drug resistance mechanisms create targetable proteostatic vulnerabilities in Her2+ breast cancers
Source: PLoS One. 2022 Dec 8;17(12):e0256788. doi: 10.1371/journal.pone.0256788 (PMC9731458; doi:10.1371/journal.pone.0256788)
Supplement: Attachment — Submitted filename: Rebuttal PLoS ONE.docx [file pone.0256788.s007.docx]

**Rebuttal**

Reviewer #1: In the era/context of precision medicine, oncogenic kinase inhibitor is the key for drug matching with specific oncogenic mutations but their response often short lived due to development of clinical (ad inito or acquired ) resistance. In this aspect this is an interesting/important article.

Critical comments:
1. This is a question to authors: Can this proteostatic vulnerabilities correlate well with immune vulnerabilities following the treatment of ant-HER2 therapies? Since authors demonstrated some correlation between proteostatic and genomic vulnerabilities.

***Response****: This is an important question. Some of our internal observations have suggested an increased expression of MHC-I expression and some pro-inflammatory response phenotype. Guido Kroemer’s group has published extensively on the role of ER stress in the innate response involving CRT exposure, and also tying genomic instability to anti-tumor innate response through ER stress. So, although the connection between the two phenotypes is certain, we have not explicitly investigated the correlation between different proteostatic phenotypes with the immune response phenotypes in tumor cells.*

2. Authors performed some experiments with BT474 cells (along with SRBR3 cells), both cells are HER2 amplified, but SKBR3 is ER negative and BT474 is ER+ cells. In their hands both behave almost similarly following the development of lapatinib resistance. Any comments from authors side about the role of ER in the development of lapatinib-induced metabolic resistance.

***Response:*** *The role of the estrogen receptor in the resistance to anti-Her2 therapy has been well-documented in both the experimental and clinical settings, perhaps due to the signaling cross-talk between the two pathways. Our study focused on the downstream of Her2 reactivation after the acquisition of resistance, seeking to exploit the ensuing metabolic vulnerabilities. Although we did not ask that question in this study, whether or not ER has a direct role in the development of the metabolic profile during chronic lapatinib/trastuzumab exposure is a good question, and perhaps should be followed up upon in future studies.*

3. Authors may provide NRG1 and EGF Western blot expression data.

***Response:*** *We have now added the NRG1 western blot in Fig.2A.*

4. In Figure 4A, please make it bold for UDP-glucose (in the figure).

***Response:*** *Done.*

5 In line 290, where authors mentioned increased citrate expression, here authors failed to mention about the high expression of Creatin Phosphate.

***Response:*** *We have now mentioned this observation in the same paragraph, along with a potential explanation.*

6. Authors may provide qRT-PCR data of GADD34 mRNA expression.

***Response:*** *We are now providing that in the supplementary materials.*

7. Please mentioned number of patients sample for Figure 5H. These patients were exposed with trastuzumab but not lapatinib. Any thoughts from authors side?

***Response:*** *We indicated the total number of samples in the figure legend for Fig.5. Unfortunately, similar data for lapatinib -treated patients do not exist in the public domain, to our knowledge. However, we believe that the mechanisms of acquired, and even de novo, resistance are similar between different anti-Her2 therapy modalities, often involving the reactivation of oncogenic tyrosine kinase signaling converging on the resumption of protein synthesis.*

Minor comments:
1. Authors failed to mentioned about the phosphorylation sites of AKT following lapatinib treatment. Better to provide both Ser473 and Thr308 phosphorylation expression.

***Response:*** *This is Ser-473 site on Akt, which is frequently used to monitor Akt activation in immunoblotting.*

Reviewer #2: The Authors, in their work, using transcriptomic, metabolomic and proteomic analysis, characterized the mechanism of acquisition of drug resistance in Her2+ breast cells. The topic of the manuscript is attractive and very important; however, some points need clarification:
• Transcriptomics and qPCR methods are not described in the material and methods section.

***Response:*** *This has now been corrected.*

• The raw data of transcriptomic and metabolomic analysis should be registered in the public repository, and the accession ID should be displayed in the main text. Moreover, it would be helpful to include the results of these analyses (lists of genes and metabolites with most altered expression) as tables in supplementary materials.

***Response:*** *We are now providing the complete RNAseq dataset of gene-level measurements in the Supplementary Table 2.*

• The results chapter should be divided into subsections that would make this chapter clearer.

***Response:*** *Done*
• There are no units in figures 1B, 2C, 2E, 3B, 3C, 4C, 4E, and supplementary results.

***Response:*** *These have now been properly annotated either on the figure axis, or in the legend.*

• The Authors should explain how the relative viability and growth rate were counted (presented in figures).

***Response:*** *We have now described this in the corresponding figure legends.*

• What was the reference gene used to normalize the qPCR results?

***Response:*** *GAPDH*

• The discussion in this publication is too general. The Authors should discuss their results in the context of other published work instead of just repeating the description of their results.

***Response:*** *We disagree that the Discussion section is “just repeating the description” of our results. The discussion section is made of 4 paragraphs. In the first, we give an overview of the “adaptive kinome reprogramming” concept that has emerged from the past studies on acquired drug resistance, and how our findings align with this concept. We also discuss how these findings have not yielded any progress in the clinical care of Her2+ breast cancer patients. In paragraph 2, we discuss a new concept of exploiting the vulnerabilities of the resistance cell state for therapeutic purposes, and give a multitude of examples from past studies, including a discussion of our own prior study in this direction in paragraph 3. Finally and only in paragraph 4, we give an overview of the results in this study, and finish with a discussion of how these results tie to the concept presented in paragraph 2, with a potential to create new therapeutic paradigms in Her2+ breast cancer patients.*

• There are some grammar and spelling mistakes.

***Response:*** *Corrected the best we could.*
